# Selective cytotoxic and anti-metastatic activity in DU-145 prostate cancer cells induced by *Annona muricata *L. bark extract and phytochemical, annonacin

**DOI:** 10.1186/s12906-020-03130-z

**Published:** 2020-12-10

**Authors:** Kimberley Foster, Omolola Oyenihi, Sunelle Rademan, Joseph Erhabor, Motlalepula Matsabisa, James Barker, Moses K. Langat, Amy Kendal-Smith, Helen Asemota, Rupika Delgoda

**Affiliations:** 1 Natural Products Institute, University of the West Indies, Mona, Kingston 7, Jamaica; 2 Biotechnolgy Centre, University of the West Indies, Mona, Kingston 7, Jamaica; 3 Pharmacology Department, School of Clinical Medicine, Faculty of Health Sciences, University of the Free State, Bloemfontein, South Africa; 4 School of Life Sciences, Pharmacy and Chemistry, Kingston University, Penrhyn Road, Kingston-upon-Thames, Surrey, UK; 5 Jodrell Laboratory, Department of Natural Capital and Plant Health, Royal Botanic Gardens, Kew, Richmond, TW9 3DS UK; 6 Faculty of Biological Sciences, University of Leeds, Leeds, England

**Keywords:** ROS, Caspase, Prostate cancer, Annonacin, Ethnopharmacology, Antiangiogenesis, Docetaxel, *Annona muricata* L

## Abstract

**Background:**

*Annona muricata* L. was identified as a popular medicinal plant in treatment regimens among cancer patients in Jamaica by a previously conducted structured questionnaire. Ethnomedically used plant parts, were examined in this study against human prostate cancer cells for the first time and mechanisms of action elucidated for the most potent of them, along with the active phytochemical, annonacin.

**Methods:**

Nine extracts of varying polarity from the leaves and bark of *A. muricata* were assessed initially for cytotoxicity using the MTT (3-(4,5-dimethylthiazol-2-yl)-2,5-diphenyltetrazolium bromide) assay on PC-3 prostate cancer cells and the ethyl acetate bark (EAB) extract was identified as the most potent. EAB extract was then standardized for annonacin content using High-performance Liquid Chromatography - Mass Spectrometry (HPLC-MS) and shown to be effective against a second prostate cancer cell line (DU-145) also. The mode of cell death in DU-145 cells were assessed via several apoptotic assays including induction of increased reactive oxygen species (ROS) production, reduction of mitochondrial membrane potential, activation of caspases and annexin V externalization combined with morphological observations using confocal microscopy. In addition, the potential to prevent metastasis was examined via inhibition of cell migration, vascular endothelial growth factor (VEGF) and angiogenesis using the chorioallantoic membrane assay (CAM).

**Results:**

Annonacin and EAB extract displayed selective and potent cytotoxicity against the DU-145 prostate carcinoma cells with IC_50_ values of 0.1 ± 0.07 μM and 55.501 ± 0.55 μg/mL respectively, without impacting RWPE-1 normal prostate cells, in stark contrast to chemotherapeutic docetaxel which lacked such selectivity. Docetaxel’s impact on the cancerous DU-145 was improved by 50% when used in combination with EAB extract. Insignificant levels of intracellular ROS content, depolarization of mitochondrial membrane, Caspase 3/7 activation, annexin V content, along with stained morphological evaluations, pointed to a non-apoptotic mode of cell death. The extract at 50 μg/mL deterred cell migration in the wound-healing assay, while inhibition of angiogenesis was displayed in the CAM and VEGF inhibition assays for both EAB (100 μg /mL) and annonacin (0.5 μM).

**Conclusions:**

Taken together, the standardized EAB extract and annonacin appear to induce selective and potent cell death via a necrotic pathway in DU-145 cells, while also preventing cell migration and angiogenesis, which warrant further examinations for mechanistic insights and validity in-vivo.

## Background

Small molecular secondary metabolites found expressed in plants have played a key, adaptive role aiding in their evolution from single cellular organisms coping in a harsh chemical soup, to being multicellular, terrestrial organisms, equipped to gain reproductive vantages or vade off over-grazers and diseases [1]. These vastly diverse group of small molecules that provide the plant with such advantages other than their primary functions of respirations, have inspired man-kind to experiment on plants over millennia for solutions to their own health problems. Thus, the high reliance on plant remedies by over 80% of the developing world for primary care [2], similar to the 73% self-medicating rates with herbs in Jamaica [3], provide evidence for the strong belief in the healing properties resident in plants. The development mean of approximately 32% of pharmaceuticals and botanical mixtures derived directly from or inspired by natural products over the past 39 years [4] for the treatment or prevention of multiple health issues including cancer, diabetes and microbial infections, provide credence to such beliefs.

Unsurprisingly plants have been utilized in the management of cancer since time immemorial in many traditional medical systems and remain a major source for bioprospecting [5], having inspired over 50% of cancer drugs approved over the past four decades [4], including vincristine, vinblastine, paclitaxel, camptothecin and podophyllotoxin [6]. Jamaica has a wide array of self-medicated herbs and medicinal plants in use against illnesses [7], with some displaying anti-cancer properties. *Petiveria alliacea* and key phytochemical, dibenzyl trisulfide [8] and the Jamaican ball moss (*Tillandsia recurvata* L.) [9] exemplifies use in prostate cancer, among other biodiversity with disparate cytotoxic properties [10]. A recent survey among cancer patients in the country [11] helped identify common ethnomedical practices in the island nation and *Annona muricata* L. emerged as a popular ingredient, in line with findings emerging from Indonesia, [12] and Trinidad [13].

Comprehensive ethnobotanical studies of the Annonaceae family have been conducted in recent years [14] and the therapeutic potential of *Annona muricata*, the most prominent species of the Annonaceae family examined in the treatment of insomnia, rheumatism, hypertension and various cancers [15]. Reports have emerged from Nigeria [16], Mexico [17] and the Philippines [15] for the therapeutic application of a decoction of the leaves to treat cancers of the prostate and stomach among others, while in Peru [18], infusions of the leaves are used for cancer treatment [15]. Evaluating these reports highlight the fact that although leaf preparations have undergone some scrutiny, bark extracts have remained largely unexplored.

The two major classes of phytochemicals found in *A. muricata* are flavonoids and acetogenins both of which are associated with a plethora of pharmacological activities individually or synergistically in a wide array of plants [19–21]. Annonacin commonly occurs in various species of the Annonaceae family and is the major acetogenin of *A. muricata* [22]. Multiple studies have demonstrated its ability to exert anti-tumor effects against endometrial, breast and skin cancer through cell cycle arrest and other cell signaling pathways [23–25]. There is mounting evidence to support the antitumor activity through apoptosis induction in numerous cancer cell lines such as colon and breast cancer [5]. This, along with cell cycle arrest at G1 phase are some of the well reported antitumor mechanisms of *A. muricata* leaf [26–30]. Though, many studies have shown mitochondrial mediated apoptosis, cell death can occur independently of mitochondrial involvement without the generation of Reactive Oxygen Species (ROS) to trigger apoptosis, and full evaluations are required in each type of cancer cell.

In this study we evaluated, the cytotoxic value of polar and non-polar leaf and bark extracts of *A. muricata,* the two most popularly used plant parts in ethnomedicine [11] on prostate cancer cells. With the aid of a panel of biochemical monitors, we demonstrate the usefulness of the most potent of those extracts, along with the key phytochemical annonacin, alone and in combination therapy with a standard chemotherapeutic drug, docetaxel. Their impact on prostate cells was independent of ROS, caspases activity and appeared to follow a necrotic pathway of cytotoxicity. Demonstrating strong anti-angiogenetic properties these natural products warrant future in-depth scrutiny on in-vivo prostate cancer models.

## Methods

### Plant material

Aerial parts (leaf and bark) of *Annona muricata* were collected in August 2017 from the Botanical Gardens at the University of the West Indies (U.W.I), Mona, Jamaica. *A. muricata*, which grows wildly in Jamaica is not an endangered species, hence no special governmental permission was required for collection. Voucher specimens were deposited at the Herbarium in the Department of Life Sciences, U.W.I., Mona with Accession Numbers 36,362 and 36,363, following authentication by herbarium curator, Mr. Patrick Lewis.

### Preparation of extracts

The leaves and bark of *A. muricata* were collected, cleaned, dried at room temperature and pulverized into powder. 5 g of leaf and bark separately or 2.5 g leaf and 2.5 g bark combined were then sequentially extracted with hexane, ethyl acetate and ethanol (200 mL each) for 3 days at room temperature. Three extracts per solvent (hexane, ethyl acetate and ethanol) were prepared using the leaf, bark and leaf:bark combination in a 1:1 ratio totaling nine extracts of varying polarity. The resulting suspension from solvent extract was filtered through type 2 Whatman filter paper and the filtrate evaporated to dryness using a rotary evaporator at low temperature [28]. The extracts obtained from each solvent were weighed, labeled and stored at − 20 °C in sealed tubes until further use.

### Cell culture

PC-3 and DU-145 human prostate carcinoma cells and RWPE-1 normal prostate epithelial cells obtained from American Type Cell Collection (ATCC, Manassas, VA, USA) were used for the cytotoxicity determination in the study. PC-3 cells were maintained in Kaighn’s modification of Ham’s F-12 medium (F-12 K) and DU-145 cells were maintained in ATCC formulated Eagle’s Minimum Essential Medium (EMEM) supplemented with 10% fetal bovine serum (FBS) while RWPE-1 cells were maintained in Keratinocyte Serum Free Medium supplemented with human recombinant epidermal growth factor and bovine pituitary extract. All cell lines were incubated in a humidified atmosphere with 5% carbon dioxide in the air at 37 °C until 90% confluence after which they were harvested for the viability experiments.

### Cell viability assay

The MTT assay (3-(4,5-dimethylthiazol-2-yl)-2,5-diphenyltetrazolium bromide) was used to evaluate cell viability. An optimized cell concentration 15,000 cells/well were seeded in 96-well plates and incubated for 24 h. Cells were subsequently treated with extracts, annonacin, docetaxel and a combination of EAB extract and docetaxel at different concentrations ranging from 50 to 100 μg/mL extract and 0.0001–0.0016 μg/mL for docetaxel and incubated for 72 h, after which the MTT solution was added and incubated for another 4 h at 37 °C. The medium was aspirated and the crystals formed solubilized with the addition of 100 μL dimethyl sulfoxide (DMSO) to each well. Finally, the resulting absorbance was measured at 570 nm using a microplate reader and the percentage of cell viability calculated as a ratio of untreated cells in vehicle control (1% DMSO). The experiments were performed in triplicate and the results expressed as the mean ± standard error of the mean [31, 32].

### Standardization of extract using HPLC-MS

5.7 mg of the ethyl acetate extract was dissolved in 57 μL methanol and analyzed by HPLC-MS (Velos-Pro, Thermo Fisher Scientific); Phenomenex C18 column: 150 × 3 mm, 3 μm particle size (method:0–1 min = isocratic gradient 10%methonol, 90% H_2_O; 1–18 min = linear gradient 30% methanol, 70% H_2_O to 78% methanol, 22% H_2_O; 18–20 min = 78% methanol, 22% H_2_0 to 100% methanol) alongside standard of pure annonacin. Concentrations were estimated from the peak area of the corresponding molecular ion peak ([M + H]^+^; m/z 597.60) in positive electron spray ionization mode, using known concentrations of annonacin standard for calibration, and accounting for dilution in 80% methanol [33]. ^1^H NMR spectrum of pure annonacin was analyzed using a 400 MHz NMR Avance spectrophotometer to authenticate it.

### Reactive oxygen species assay

The generation of reactive oxygen species (ROS) is often associated with chemotherapeutic and other non-surgical interventions in cancer, as a means of triggering cell death. Often the intracellular generation of ROS is indicative of early induction of apoptosis [34]. Since ROS generations were previously observed for *A. muricata* leaf and twig extracts in HL-60 leukemia cells [35], we evaluated such potential by EAB extract and annonacin on DU-145 cells. Intracellular ROS generation was determined using the single reagent 2′,7′– dichlorofluorescein diacetate (DCFDA). This is a cell permeant, fluorogenic dye which can be oxidized to DFC (2′,7′– dichlorofluorescein) as the fluorescent product, detected by a spectrofluorometer used to measure hydroxyl, peroxyl and other reactive oxygen species (ROS) activity within the cell [35]. DU-145 cells were treated with the EAB extract, annonacin and 2.5 μM hydrogen peroxide (H_2_O_2_) as positive control for 72 h. After incubation, the media was removed, cells were washed with Phosphate-buffered saline (PBS) then stained with 100 μL DCFDA and incubated for another 30 mins in the dark at 37 °C. After which the fluorescence intensity was subsequently measured at 485 nm excitation and 535 nm emission using a microplate reader.

### Mitochondrial membrane potential using the JC-10 assay

The mitochondria play an important role in apoptosis detection and induction of cell death [36]. The change in mitochondrial membrane potential (MMP) was measured using the JC-10 assay kit (Sigma-Aldrich, USA) according to vendor’s instructions. This uses a dual emission fluorescent dye capable of entering the mitochondria and emits either red or green fluorescence depending on the state of the mitochondrial membrane. Red fluorescence is seen in normal polarized mitochondria while a green fluorescence is obtained when the mitochondrial membrane potential decreases and the membrane is depolarized causing the dye to diffuse into the cytoplasm of the cell. Treated DU-145 cells (with docetaxel, varying concentrations of EAB, annonacin, control and 10 μM of H_2_O_2_) were incubated in 96-well plates for 72 h. After incubation, the media was removed, cells were washed with PBS then stained with 50 μL/well of the JC-10 dye loading solution. The plate was then incubated for 50 mins in a 37 °C incubator protected from light after which 50 μL/well of assay buffer B was added, then fluorescence intensity subsequently read at excitation/emission wavelengths of 490/525 nm and 540/590 nm [37]. Results are recorded as a ratio of red to green fluorescence.

### Human Annexin V assay

During apoptosis, the cell membrane is altered and Phosphatydylserine (PS) located in the membrane leaflets become exposed at the cell surface and allow for binding of annexin V. Total annexin V in treated DU-145 prostate cancer cells was quantitatively measured with the Human annexin V Platinum ELISA kit (Affymetrix, eBioscience, Vienna, Austria) by comparing to the standard provided in kit following the vendor’s kit manual. Briefly, 50 μL of treated cell supernatant in triplicates was used for annexin V determination. The assay was conducted at room temperature and results monitored at 620 nm [38].

### Caspase 3/7 assay

Manufactures’ instructions were followed for CellEvent® Caspase-3/7 Green reagent allowing detection of Caspase 3 activity. Briefly, treated DU-145 cells were incubated for 24 h with 0.6 μg/mL annonacin, (50 and 100 μg/mL) EAB extract and 10 μM of H_2_O_2_ as positive control in 96-well plates at a concentration of 15,000 cells/well. After incubation 4 μM Caspase-3/7 Green Detection Reagent was added to each well and incubated for 30mins. The results were analyzed by a fluorescence microplate reader at 503 nm/530 nm excitation/emission [39, 40].

### Ethidium bromide and Acridine Orange staining

Cells were seeded into 6-well plates lined with coverslips at a concentration of 250,000 cells/well. Confluent cells were treated with various concentrations of extract and compound and incubated for 72 h. After incubation, the cells were subsequently washed with PBS and then treated with a dye mixture containing ethidium bromide and acridine orange (1:1, 100 μg/mL) for 15 min covered with foil. After 15 mins the stain was removed, and the cells rinsed with PBS. 1 ml of paraformaldehyde was added to the cells for 15 mins to fix the stained cells on the coverslips. After removing the paraformaldehyde, the coverslips were removed and mounted on slides viewed under confocal microscopy [41].

### Cell migration assay

The more metastatic prostate cancer cell line (PC-3) was used to assess the anti-metastatic potential of the EAB extract. PC-3 cells were seeded into 6-well plates at a concentration of 250,000 cells/well. Confluent cells were scratched using a sterile 200-μl pipette tip and washed twice with PBS to remove detached cells. The image of cells in each well was captured at time 0 h. Cells were treated with various concentrations of extract and incubated for 24 h at 37 °C with 5% carbon dioxide in the atmosphere. After incubation, cells were washed with PBS to remove cell debris [28, 42]. Images were subsequently captured after incubation using an Amscope digital eyepiece microscope camera attached to an inverted microscope. Data was statistically analysed using GraphPad software, and results were  expressed as percentage cell migration.

### Chicken chorioallantoic membrane - CAM assay

Three eggs per sample (in triplicates) were obtained from a local hatchery in Bloemfontein, South Africa and incubated for 8 days at 37 °C with 60% humidity. Egg shells were sterilized with 70% ethanol and a 1 cm^2^ window on the air space end was cut opened on the 8th day to expose the blood vessels. A 1 cm^2^ sterile Whatman filter paper shocked with the compound, extract, Tinzaparin (positive control) and L-arginine (negative control) was placed on the surface of the growing CAM vessels. The eggs were then labelled and re-sealed with sterile adhesive tape in a laminar flow hood and incubated for another 3 days. On day 11, the CAMs were reopened in sterile petri dishes, photographed and blood vessels in each CAM were counted. The results were presented as the angiogenic index for each sample [43, 44].

### VEGF inhibition assay

The extracellular vascular endothelial growth factor (VEGF) levels were assayed using the supernatant of earlier treated DU-145 cells [43]. The cells were seeded in a 48-well plate at a concentration of 50,000 cells per well. To influence the cell growth and VEGF production, 0.01 mg/ml of insulin was supplemented in the culture medium. After 24 h incubation, cells were treated with extract and test compound and incubated for 72 h after which plates were centrifuged at 5000 g for 10 min and the supernatant collected for VEGF estimation. Total VEGF content in cultured supernatants was estimated following the instructions of Human VEGF ELISA kit (ThermoFisher Scientific).

### Statistical analysis

The results were expressed as the mean ± standard errors of the mean. Assays were conducted in three individual experiments, each performed in triplicates. IC_50_ values were determined using nonlinear regression analysis on Sigma Plot (version 10.0) software. All other statistical analyses were performed with GraphPad Prism 8.0 (USA). The overall effects of plant part, solvent type and the interaction of both on cell viability were determined using two-way ANOVA followed by Tukey’s multiple comparisons test to check for significant differences between the data. One-way analysis of variance (ANOVA) followed by Tukey’s multiple comparisons post hoc test was used to compare treated cells with the control. Significant differences were reported with *** indicating a *p*-value < 0.0001, ** indicating a *p*-value < 0.001 and * indicating a *p*-value < 0.05.

## Results

### Selective cytotoxic effect of *A. muricata* extracts on PC-3 prostate cancer cells and RWPE-1 normal prostate cells

**Fig. 1:**
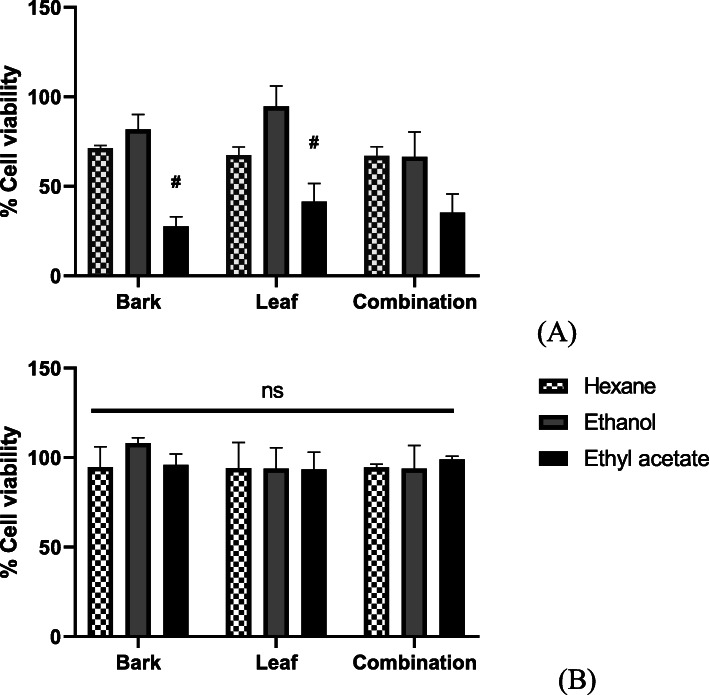
*A. muricata L*. leaf and bark extracts display selective anti-proliferative activity on prostate cancer cells. PC-3 prostate cancer cell line (**a**) and RWPE-1 normal prostate cell line (**b**) were treated for 72 h with 100 μg/mL extracts of hexane, ethanol and ethyl acetate of leaf, bark alone and leaf:bark in 1:1 ratio. Cell viability was calculated as a percentage of solvent control (1% each solvent as control for each type of extract) and results are represented as mean ± SEM (*n* = 3). For different extracts with the same plant part, # represents statistically significant differences (*p* < 0.05, two-way ANOVA). ‘ns’ represents no statistical significance

Nine extracts of varying polarity including three extracts per solvent (hexane, ethyl acetate and ethanol) prepared using the leaf, bark and leaf:bark combination in a 1:1 ratio were analyzed for their cytotoxic potential. This experiment utilized the most commonly used parts of the plant in ethnomedical practices. All such extracts prepared were subsequently screened at a concentration of 100 μg/mL against cancerous (PC-3) and normal (RWPE-1) prostate cell lines and results depicted in Fig. 1. Inducing the largest impact (30% cell viability) on the cancerous PC-3 cells, the ethyl acetate extract of the bark was identified as the most potent and selective, with negligible impact (> 90% cell viability) on the normal cells. It is noteworthy that all examined extracts of this plant elicited low cytotoxicity on normal cells.

### Standardization of ethyl acetate bark extract

**Fig. 2:**
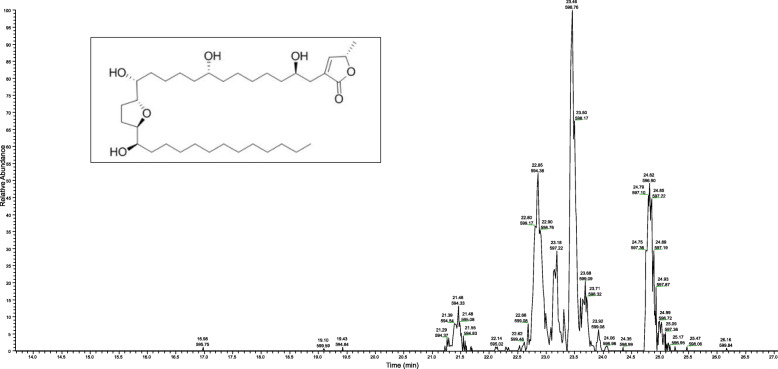
HPLC-MS chromatogram of the standardized EAB extract showing annonacin. Filtered positive mode output (597.5 (+/− 1.5) from Velos pro representing the annonacin (inset) abundance in bark of *Annona muricata.* Annonacin is identified at 23.18 min

Having identified the ethyl acetate extract of the bark (EAB) as the most potent, the presence of the key phytochemical, annonacin, was identified and quantified in this extract using HPLC-MS (Fig. 2). The level of annonacin was < 100 ppm when compared to its standard. After filtering for a molecular weight of 597.5 (+/− 1.5) in positive mode, accounting for the added ion to annonacin molecular weight, and compared to the standard it was determined that annonacin is identifiable with a retention time, leaving the column at, 23.18 min with a molecular weight of 597.60.

### Improved cytotoxicity of chemotherapeutic drug, docetaxel in combination with standardized EAB extract

**Fig. 3:**
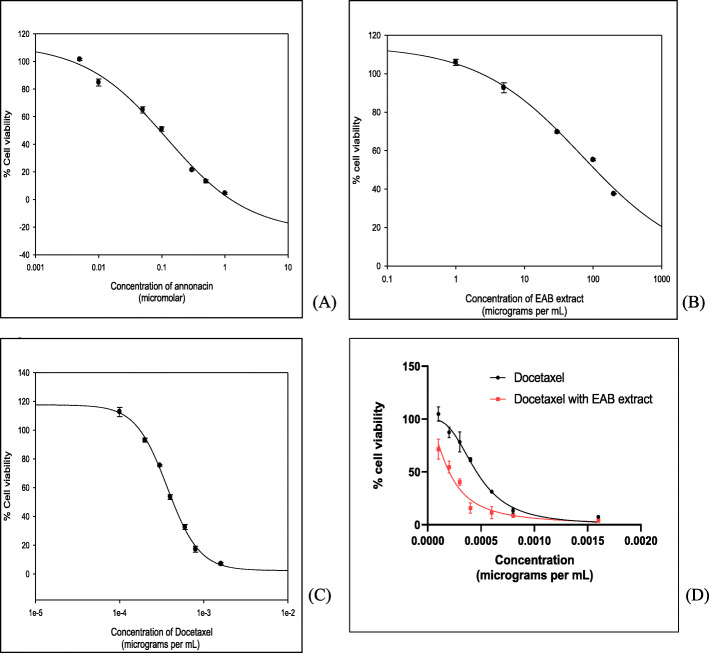
EAB extract and annonacin display dose dependent impact on cell viability of DU-145 cells. Antiproliferative activity of (**a**) annonacin, (**b**) ethyl acetate extract of *A. muricata* bark (EAB), (**c**) docetaxel and (**d**) docetaxel in combination with 100 μg/ml EAB extract against DU-145 prostate cancer cells determined by MTT assay after 72 h incubation with varying concentrations of each sample. Results are means ± SEM of triplicates in three independent experiments and the percentage of cell viability was calculated as a percentage of solvent control (1% DMSO). A dose dependent decrease in cell viability was observed with all test samples and combining docetaxel with the EAB extract reduced the observed IC_50_

To ensure that the observed cytotoxicity with PC3 in Fig. 1 was not cell line dependent, we examined the effect of EAB extract and annonacin on DU-145 cells also, a cell line derived from brain metastasis of human prostate cancer [45, 46]. Dose dependent inhibitions of the growth of DU-145 were observed after 72 h incubations (Fig. 3) and the IC_50_ values obtained for the extract (55.5 ± 0.55 μg/mL) and annonacin (0.079 ± 0.07 μg/mL or 0.1 μM), were compared to that of docetaxel (0.0004 ± 1.59X10^− 5^ μg/mL or 0.05 nM), a standard chemotherapeutic drug as shown in Table 1. Combining docetaxel with EAB extract induced an even greater impact on cell viability (Fig. 3d), reducing the IC_50_ of the former to 0.0002 μg/mL within a 95% confidence interval. A likely synergistic interaction underlies this improvement of docetaxel impact in the presence of the extract and we recommend future studies for a full understanding of this hypothesis. Having observed that the EAB extract is effective in reducing the cell viability of a second type of prostate cancer cell line, we undertook further work on the extract to gain mechanistic insights using DU-145 cells. Since DU145 models a moderately metastatic prostate cancer as opposed to grade IV adenocarcinoma PC3 cells with high metastatic potential, we selected the DU145 cell line for mechanistic study with suitability for studying treatment interventions in the early stages. Additionally, PC3 is suspect of carrying co-regulators for tumor suppression, which could complicate mechanistic studies, weighing into our decision to work with DU145 cells for this study.

**Table 1:** IC_50_ values for the extract, annonacin and docetaxel on cancerous DU-145 cells and normal RWPE-1 cells, after 72 h

Treatment	Cell lines, IC_50_ value (μg/mL)	
	DU-145	RWPE-1
EAB extract	55.501 ± 0.55	> 300
Annonacin	0.0793 ± 0.07	> 0.48
Docetaxel	0.0004 ± 1.59	0.0004 ^α^

Results are expressed as mean ± SEM of three independent experiments. ^α^- IC_50_ value obtained from Karanika et al. [47]

### EAB extract and annonacin does not elicit ROS generation in DU-145 cells

**Fig. 4:**
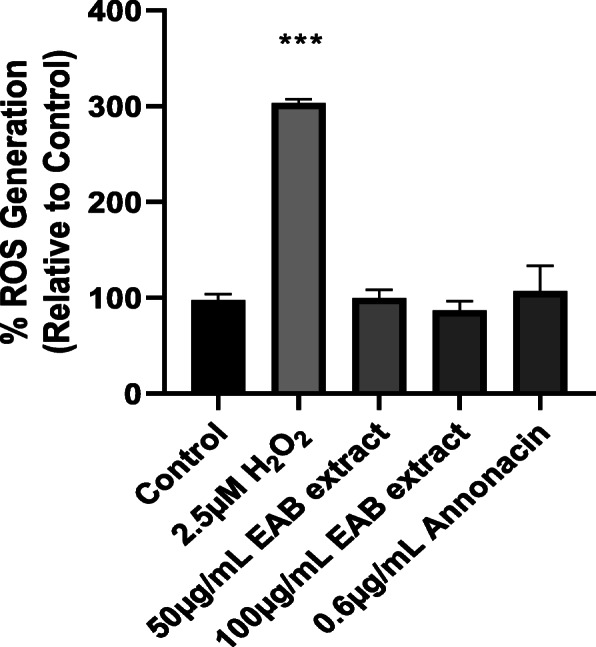
EAB extract and annonacin yield no impact on intracellular Reactive Oxygen species (ROS). Intracellular ROS generated in DU-145 cells treated with 2.5 μM H_2_O_2_ (positive control), extract and annonacin relative to untreated cells (control) were assessed after 72 h using the fluorescent probe DCF-DA. EAB extract and annonacin showed no impact on ROS generation. Data are means ± SEM of triplicates in three independent experiments. Statistical significance indicated by *** (one-way ANOVA, *P* < 0.0001)

Our results using the fluorogenic probe, 2, 7-dichlorofluorescin diacetate (H_2_DCFDA) which oxidizes to its highly fluorescent form dichlorofluorescein (DCF) in the presence of ROS, indicate that neither the *A. muricata* extract nor annonacin triggered significant increases in levels of intracellular ROS in prostate cancer cells. When compared to the untreated control, there was no difference in the percent ROS obtained as shown in Fig. 4, in contrast to a 2.5 μM solution of hydrogen peroxide which elicited a three-fold increase in oxidative capacity, while 10 μM hydrogen peroxide elicited a nineteen fold increase (data not displayed).

### Measurement of mitochondrial membrane potential by JC-10 assay

**Fig. 5:**
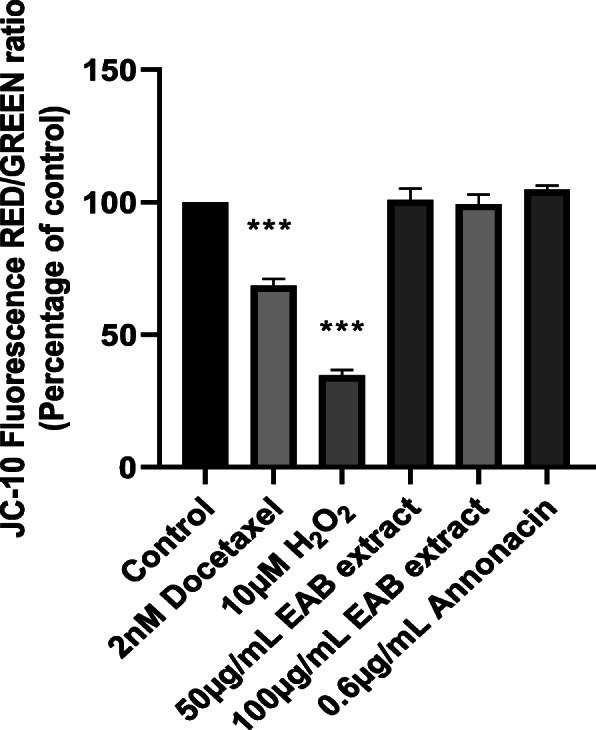
EAB extract and annonacin does not alter the mitochondrial membrane potential (MMP) in DU-145 cells. Quantitative evaluation of the effects of annonacin and EAB extract on MMP in DU-145 cells after 72 h was carried out using JC-10 Fluorescence dye computed relative to untreated cells (control). Cell were treated with 10 μM H_2_O_2_ and 2 nM docetaxel as positive control as docetaxel disrupts MMP. EAB extract and annonacin displayed healthy cell content of near 100% similar to the control, while those treated with docetaxel yielded 69% healthy cell and hydrogen peroxide displayed 39% healthy cell ratio, as measured by the healthy (red) cell count in comparison to the damaged (green) cells. . Data are means ± SEM of triplicates in three independent experiments. Statistical significance indicated by *** (one-way ANOVA, *P* < 0.0001)

Cells treated with annonacin and EAB extract displayed a ratio comparative to the control healthy cells, indicating that the cytotoxic effect of *A. muricata* bark extract in prostate cancer cell does not involve the depolarization of mitochondrial membrane (Fig. 5). In contrast, docetaxel (known to impart anti-cancer activity via apoptotic pathway) displayed significantly lower ratio of healthy red cells in comparison to the damaged green cells (69%, red:green ratio) in comparison to the untreated cells. Similarly, a 10 μM solution of hydrogen peroxide solution was able to depolarize the mitochondrial membrane resulting in a significantly reduced (35%) healthy cell content ratio in comparison to the control.

### Human Annexin V externalization confirms lowered apoptotic body formation

**Fig. 6:**
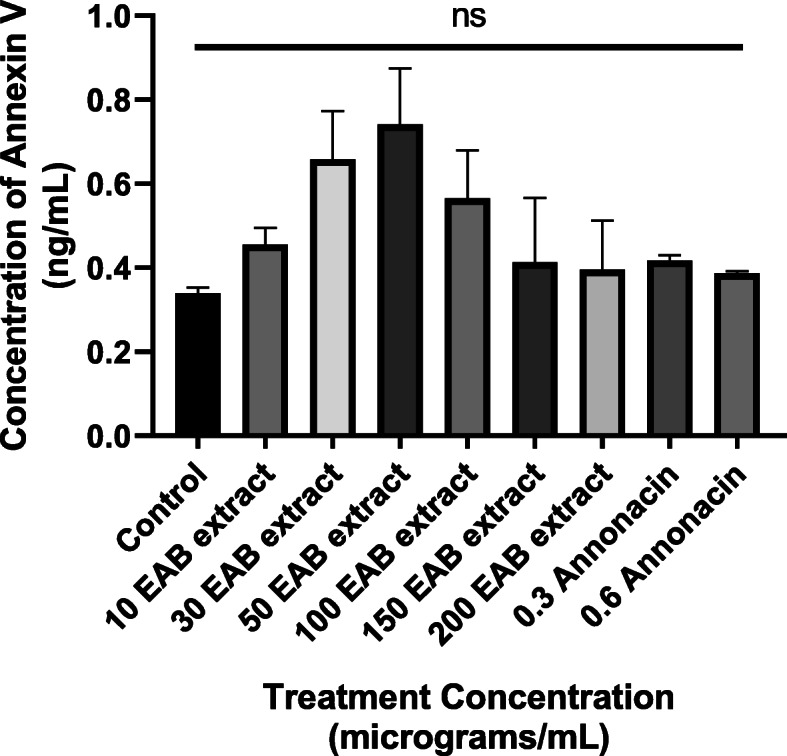
EAB extract and annonacin generates low annexin V content. Figure displays the concentrations of annexin V detected in DU-145 cells treated with varying concentrations of EAB extract and annonacin for 72 h calculated based on the standard curve generated. Data are expressed as means ± SEM of triplicate values and no statistically significant differences (indicated by ‘ns’) were observed from corresponding controls (one-way ANOVA), albeit the somewhat varying patterns displayed

To further confirm the absence of an apoptotic pathway, the levels of annexin V present in the cell membrane of treated DU-145 prostate cancer cells were quantitatively determined using the Human annexin V ELISA kit and compared to the provided standard, annexin V in buffered protein base. Results indicate that treated DU-145 cells displayed low levels of annexin V when treated with annonacin as evident in Fig. 6, with anomalous behavior at 30 μg/mL and 50 μg/mL. However, statistical analysis showed there was no significant difference between all tested concentrations in comparison to the control. Whether there is some combination of apoptotic bodies being formed at these low concentrations followed by other forms of cell death, remains to be fully explored, but certainly the Fig. 6 indicates that between the range of 10-200 μg/mL annexin V levels stayed fairly uniform. Similarly, the levels of annexin V detected when cells were treated with annonacin is uniformly low as well.

### Measurement of Caspase-3/7 activity

**Fig. 7:**
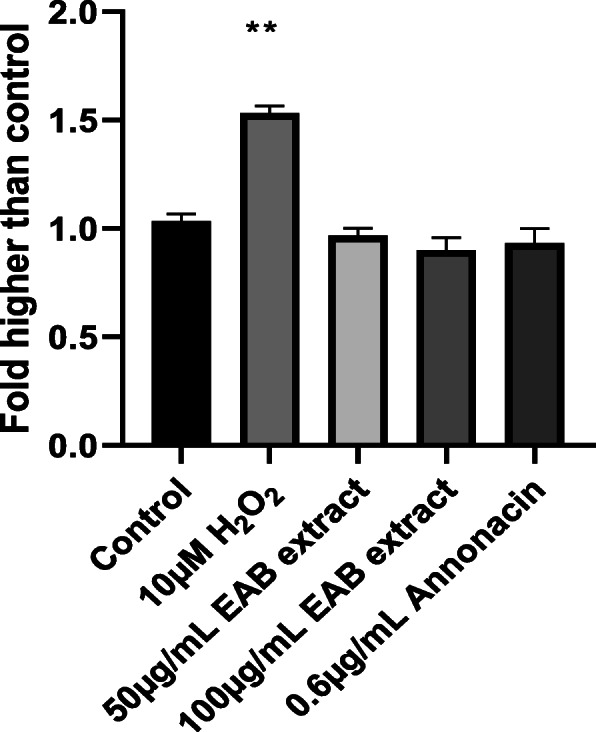
Neither EAB extract nor annonacin increases caspase 3/7 activity. Relative fluorescence expression of caspase-3/7 activity in DU-145 cells treated for 24 h with annonacin and ethyl acetate extract of *A. muricata* were detected using the fluorescence caspase-3/7 green reagent using untreated cells as control while 10 μM H_2_O_2_ was used as positive control. No caspase-3/7 was detected when cells were treated with EAB extract and annonacin as expressed in fold higher than control activity. Data are expressed as means ± SEM of triplicate values. Data subjected to analysis using one-way ANOVA. ** indicates statistically significant difference from corresponding untreated control (*P* < 0.001)

The activation of caspase-3 and caspase-7 which are major players in the caspase cascade signaling apoptotic cell death were evaluated to further characterize the cytotoxicity induced by EAB and annonacin. As seen in Fig. 7, neither annonacin nor the ethyl acetate extract of *A. muricata* bark increased caspase-3/7 activity when compared to untreated cells, suggesting a caspase independent cell death. On the other hand, an increase in caspase-3/7 activation was observed in cells treated with a 10 μM solution of hydrogen peroxide - a widely used apoptosis inducer [48].

### Acridine Orange/ Ethidium bromide staining confirm morphological changes

**Fig. 8:**
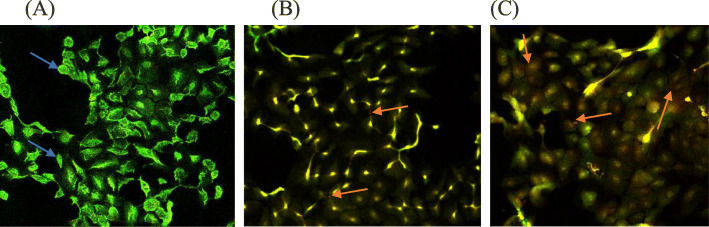
Confocal microscopy images of acridine orange/ethidium bromide double stained DU-145 cells reveals necrotic pathway in the presence of EAB and annonacin treatment. Treatment with (**b**) 50 μg/mL EAB extract and (**c**) 0.6 μg/mL annonacin for 72 h compared to (**a**) untreated cells as control reveal necrotic cells (red in appearance, red arrow) in annonacin and EAB treatment groups, in comparison to viable healthy cells as stained uniformly green (blue arrow) in (A) in the control group. Magnification 10 ×

The images obtained after double staining treated cells with acridine orange and ethidium bromide following a 72-h exposure to EAB extract and annonacin revealed morphological changes which permits qualitative detections of modes of cell death. In Fig. 8 cells in the control group appeared normal on confocal microscopy images exhibiting bright green fluorescence signals from the nuclei, suggesting the uptake of acridine orange stain [49]. In the treatment groups, majority of the cells emitted orange to red fluorescence signaling the uptake of ethidium bromide stain through damaged cell membranes. The nuclei of the cells were also characteristically uniform depicting necrotic pathway, as they did not display visible apoptotic characteristics such as fragmentation of the nuclei or formation of apoptotic bodies.

### Inhibitory effect of EAB extract on the migration of prostate cancer cells

**Fig. 9:**
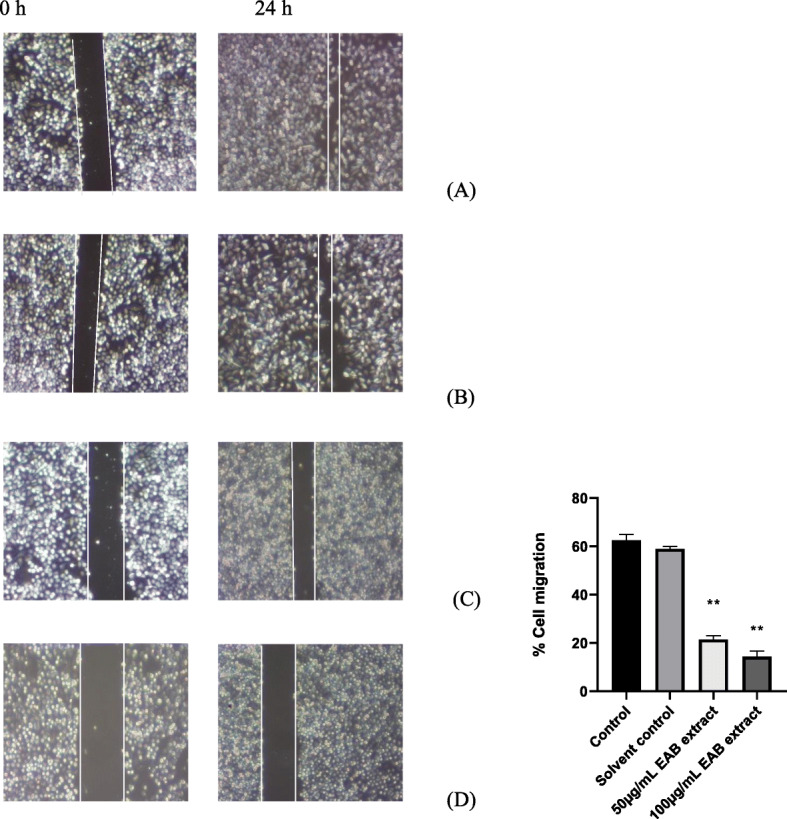
EAB extract significantly reduces cell migration. EAB extract at 100 μg/ml (**d**) and 50 μg/ml (**c**) inhibited migration of highly metastatic prostate cancer cells PC-3 cells compared to untreated control (**a**) and cells treated with solvent ethyl acetate (**b**). Cells were seeded into 6-well plates for 24 h after which each well was scratched at image captured at 0 h, treated and incubated for another 24 h where images were subsequently captured to demonstrate the extent of migration. The maintenance of a larger denude (visually) in the EAB treated groups indicate the lowered levels of cell migration (quantitated). Statistical significance indicated by ** indicating a *p*-value < 0.001 (one-way ANOVA)

In order to assess *A muricata’s* effect on endothelial cell migration, a visual depiction was garnered using an in-vitro wound healing assay on highly metastatic PC-3 prostate cancer cells as shown in Fig. 9. Both 50 μg/ml and 100 μg/ml of EAB extracts maintained a significant clearing of the denuded area created by the scratch on the monolayer of cells, in comparison to the ethyl acetate (solvent) treated and untreated controls after 24 h. Treatment yielded less than 20% of cell migration rates compared to the control groups seen to promote wound healing and cell migration for return of cell-cell contact.

### Quantification of angiogenic index induced by annonacin and EAB extract via the CAM assay

**Fig. 10:**
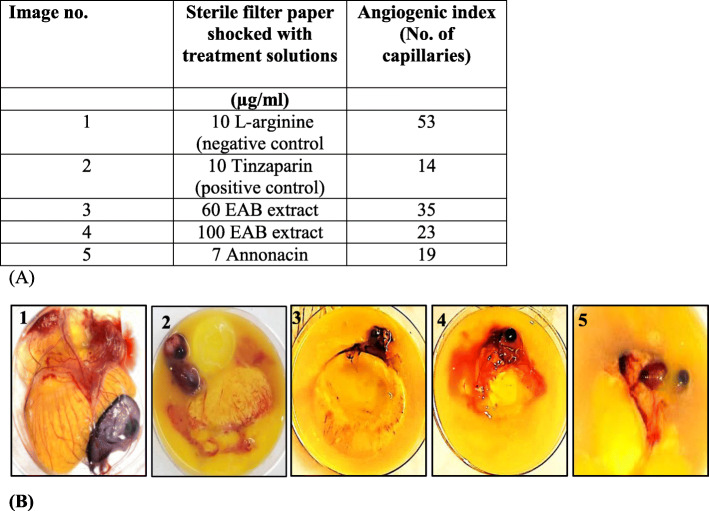
Suppression of angiogenesis by EAB extract and annonacin. Representative results of the CAM assay displaying (**a**) angiogenic index obtained from each treatment and (**b**) images of the treated eggs opened on day 11. The images represent the following treatments 1: L-Arginine, 2: Tinzaparin (20 μg/mL), 3:EAB extract (60 μg/mL), 4:EAB extract (100 μg/mL), 5: Annonacin (7 μg/mL) and a summary of the angiogenic index obtained from each treatment. The results indicate a lowered angiogenesis potential in the presence of EAB and annonacin

Further evaluations on the *A. muricata* extract on angiogenesis and tumor invasion was garnered by an assessment using the highly vascularized CAM assay. The EAB extract at 60 μg/ml and 100 μg/ml inhibited the formation of new blood vessels in the CAM with an angiogenic index of 35 and 23 respectively when compared to the negative control L-Arginine, angiogenic index 53. The suppression of angiogenesis shown by the compound annonacin was similar to that observed for the positive control tinzaparin (angiogenic index, 14). Annonacin at 7 μg/ml (11.7 μM) had the lowest angiogenic index of 19 amongst the tested samples when compared to the positive control Tinzaparin as well as the negative control L-Arginine as represented in Fig. 10 which shows less defined capillaries in images 3–5 similar to image 2 (positive control) in comparison to image 1 with well-developed capillaries. These results highlight their antiangiogenic potential which might prove beneficial in preventing cancer metastasis.

### VEGF inhibition induced by annonacin and EAB extract

**Fig. 11:**
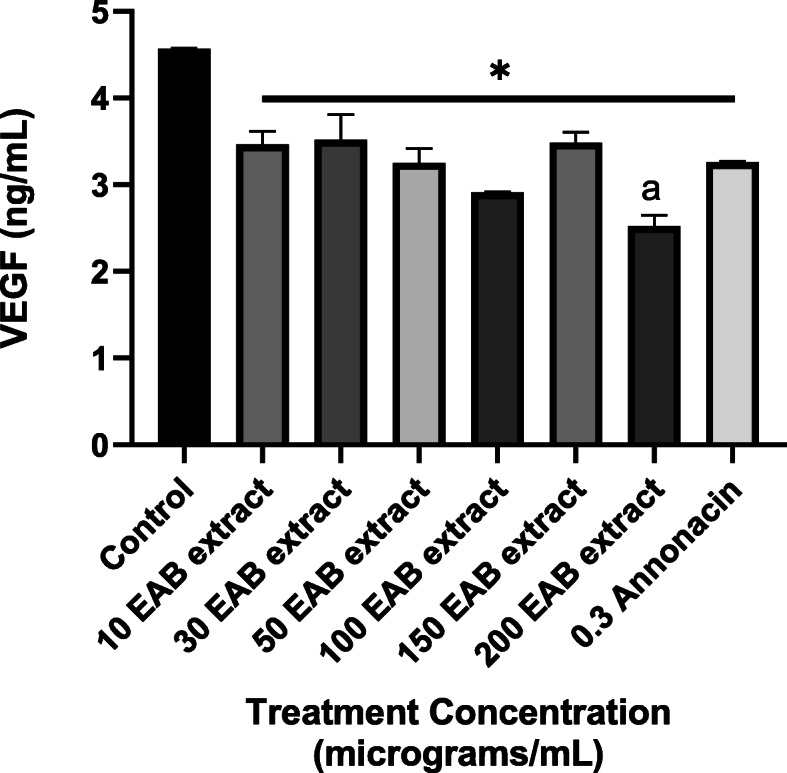
VEGF levels are significantly decreased in the presence of EAB extract and annonacin. DU-145 cultured supernatants obtained after 72 h treatment with various concentrations of EAB extract and annonacin are displayed. . Results are expressed as means ± SEM of triplicate values. * indicates statistically significant difference of all samples from corresponding control while ^a^ denotes significance between the groups (one-way ANOVA, *P* < 0.05)

To further evaluate the impact on angiogenesis, effect on a potent angiogenetic factor, Vascular endothelial growth factor (VEGF) elicited by the natural extracts were quantified. All tested concentrations of the EAB extract significantly reduced the levels of VEGF in the cell in comparison to the control. However, 200 μg/mL of the extract, had the highest inhibition against the extracellular VEGF level and was significantly lower when compared between the groups (Fig. 11). Annonacin at a concentration of 0.6 μg/mL (1 μM) was also found to reduce extracellular VEGF level when compared to the untreated control (media).

## Discussion

Recognizing the value of ethnomedicine in the search for novel solutions, this investigation evaluated extracts of *Annona muricata*, which are employed by 52% of prostate cancer patients in Jamaica as home remedies [11], for impact against prostate cancer cells. The leaves and bark of this popular fruit tree are prepared as decoctions and infusions, and thus following evaluations of nine extracts of varying polarity, the ethyl acetate extract of the bark was deemed the most potent. It was therefore standardized for annonacin content and evaluated for impact on prostate cancerous and normal cells followed by likely mechanism inducing such impact.

*A. muricata* is reportedly one of the most commonly used plants as complementary and alternative treatment by cancer patients in various cultures [11, 13, 50] and often used concomitantly with prescription medicines in patients undergoing chemotherapy [51, 52]. Interestingly, as Fig. 3 depicts, a possible synergistic interaction renders the EAB extract docetaxel combination significantly more effective than the pharmaceutical alone. Combined with the fact that annonacin and *A. muricata* extracts imparted highly selective cytotoxicity on cancerous cells in comparison to normal cells, stands it apart from pharmaceutical treatment alone. The potent IC_50_ value of 55 μg/ml for EAB extract against the cancerous cells dwarfs in comparison to its IC_50_ for the normal prostate cells (> 300 μg/ml), similar to the 6-fold increase in IC_50_ of annonacin on normal cells (Table 1), while the impact of docetaxel stands in stark contrast invoking comparable toxicity on both normal and cancerous cells [47].

Results garnered from Figs. 4 and 5 in this study, indicate that neither the extract nor annonacin induced cell death with an increasing ROS content or damage to the mitochondrial membrane. Cell deaths were observed without the activation of caspases (Fig. 7), a key requirement used to confirm the induction of apoptosis in cancer cells [53]. High doses of ROS can cause an irreversible loss of mitochondrial membrane potential in cells leading to the release of cytochrome c from the mitochondria and subsequent signaling of executioner caspases resulting in programmed cell death via apoptosis pathway [34, 54]. Most pathways of programmed cell death involve regulation by the mitochondria but there are instances where cell death is controlled in the plasma membrane by its many receptors responsible for death signaling such as tumor necrosis factor and Fas [55]. Studies have demonstrated the potential of *A. muricata* extract to inhibit TNF-α [56]. The results of the annexin V binding assay in Fig. 6 illustrates that there was some amount of phosphatidylserine exposure detected by increase in annexin V concentration around the IC_50_ which was not observed at higher concentrations of the extract. Although externalization of phosphatidylserine is characteristic of apoptotic cells, no significant increase in its content was observed and it has been shown where phosphatidylserine can be detected in early primary necrosis [57]. Other forms of programmed cell death include necrosis-like cell death characterized by the absence of both chromatin condensation and caspase activation [58].

Engagement of necrosis-like form of cell death was further suspected from the morphological observations using fluorescence microscopy following acridine orange/ethidium bromide staining (Fig. 8). Both extract and phytochemical altered the cellular morphology of the cells, exhibiting typical necrotic characteristics with the absence of chromatin condensation. Although some forms of apoptosis cannot be totally ruled out. *A. muricata* has been shown to induce necrosis in pancreatic cancer cells via the inhibition of cellular metabolism [59], typical also of some other natural products [4]. Necrosis can occur via an organized process resulting from a signaling cascade involving RIP kinase which is then termed necroptosis as some of the biochemical markers of this process are similar to apoptosis [53, 60]. The cross talk between apoptosis and necroptosis involving numerous other pathways provides an opportunity for therapeutic development that can selectively target both or certain desired avenues [61]. Although apoptosis is the cell’s preferred form of cell death, many tumors find effective ways for its evasion, leading to chemoresistance and tumor survival. Thus, therapies capable of activating non-apoptotic pathways potentially provide manipulation of cell deaths which would enhance their chemotherapeutic potential, should such resistance be developed. Whether or not the consumption of these natural products illicit an immune response as a result of resistance to apoptosis, and whether chronic inflammations is a result, are concerns worthy of future investigations using in-vivo models.

The EAB extract proved able to inhibit motility in the highly metastatic PC3 cell line preventing the wound healing process as demonstrated by the cell migration assay in Fig. 9, in addition to the significant inhibition of extracellular VEGF in Fig. 11. The EAB extract and annonacin also displayed potential in inhibiting the formation of new blood vessels in the CAM (Fig. 10). The ability of the tested extracts and annonacin to inhibit extracellular VEGF levels and blood vessel formation adduce to the probable potential of the samples to inhibit angiogenesis, one of the key mechanistic steps for tumor growth, invasion and metastasis in all cell types. Collectively, the results point to an interference in metastatic process, revealing potential of *A. muricata* in prostate cancer treatments.

## Conclusion

The present study is the first demonstration (as far as the authors are aware) of selective, potent cytotoxic effects *A. muricata* bark extracts, against prostate cancer cell lines (PC3 and DU-145) in comparison with normal cells. Via a panel of in-vitro biochemical probes, the standardized ethyl acetate extract of the bark demonstrated a necrotic path of cell death without inciting reactive oxygen species, inhibiting markers of angiogenesis and enhanced the impact of the chemotherapy docetaxel on DU-145 cells. Taken together, these findings suggest the potential of annonacin and *A. muricata* bark extract as selective cytotoxic agents with antimetastatic, antiangiogenetic potential and warrants in-vivo investigations to determine physiological measures as well as a complete understanding of the mechanism(s) of the observed cytotoxicity.

## Data Availability

The datasets used and analyzed during the current study are available from the corresponding author.

## Abbreviations

HPLC-MS: High-performance Liquid Chromatography - Mass Spectrometry
EAB: Ethyl acetate bark extract of *Annona muricata*
PC-3: Human prostate carcinoma cell line
DU-145: Human prostate carcinoma cell line
RWPE-1: Human prostate normal epithelial cell line
ROS: Reactive oxygen species
DTS: Dibenzyl trisulfide
MTT: 3-(4,5-dimethylthiazol-2-yl)-2,5-diphenyltetrazolium bromide
DMSO: Dimethyl sulfoxide
DCFDA: 2′,7′– dichlorofluorescein diacetate
H_2_O_2_: Hydrogen peroxide
PBS: Phosphate-buffered saline
MMP: Mitochondrial membrane potential
AO/EB: Acridine orange/ethidium bromide
VEGF: Vascular endothelial growth factor
CAM: Chorioallantoic membrane
